# Comparison of Carcass and Meat Quality Obtained from Mule and Donkey

**DOI:** 10.3390/ani10091620

**Published:** 2020-09-10

**Authors:** Paolo Polidori, Silvia Vincenzetti, Stefania Pucciarelli, Valeria Polzonetti

**Affiliations:** 1 School of Pharmacy, University of Camerino, Via Circonvallazione 93, 62024 Matelica, Italy; 2 School of Biosciences and Veterinary Medicine, University of Camerino, Via Circonvallazione 93, 62024 Matelica, Italy; silvia.vincenzetti@unicam.it (S.V.); stefania.pucciarelli@unicam.it (S.P.); valeria.polzonetti@unicam.it (V.P.)

**Keywords:** carcass quality, meat quality, donkey, mule, minerals, amino acids

## Abstract

**Simple Summary:**

Meat is an important source of proteins, minerals, and vitamins, and for this reason it largely contributes to the daily intakes of these nutrients in the human diet. Donkey carcass traits and donkey meat quality parameters have been determined in previous studies, while mule carcass and meat quality characteristics have never been evaluated. The aim of the present study was to compare the carcass data and meat composition obtained from 10 male donkeys and 10 male mules slaughtered at an age of 16 ± 1 years. The mules carcass weight and dressing percentage were significantly higher compared to those of donkeys. The meat quality parameters detected in both mules and donkeys showed interesting results; rumenic acid (CLA), eicosapentaenoic acid (EPA), and docosahexaenoic acid (DHA) have been detected in the lipidic profile in both meats, such as all the essential amino acids. Two important sensorial characteristics showed significant differences between the two meats examined: a darker color and higher shear force values have been detected in mule’s meat. The results obtained in this study showed that equid meat can be considered a healthy and nutritionally interesting alternative red meat.

**Abstract:**

The aim of this study was to compare the carcass characteristics and the chemical and physical parameters of the meat produced by 10 male crossbred donkeys and 10 male mules slaughtered at 16 ± 1 years of age. The carcass weight and dressing percentage were significantly (*p* < 0.05) higher in mules. Samples of the muscle Longissimus thoracis (LT) were analyzed. Donkey meat showed a higher fat content, while the glycogen content was higher in the mule LT. The total collagen content was higher in the mule LT; in this muscle, the shear force values were higher compared to the donkey LT. The lightness parameter (L*) was lower in the mule LT, while the redness index (a*) was higher in the mule LT; this muscle showed a higher content of iron, while zinc was higher in the donkey muscle LT. The donkey LT muscle showed a higher content of essential amino acids (52.2%) compared to the mule LT (50.1%). The results obtained demonstrated that the chemical and physical traits of mule and donkey meat were similar to those of other kinds of equid meat.

## 1. Introduction

Mules are the result of crosses between a male donkey and a female horse; there are estimated to be around 15 million of them in the world [1,2]. The resulting hybrid offspring from this cross show better physical and mental traits when compared with the parents due to the hybrid vigor [3].

These animals are normally slaughtered at the end of the working career; the meat obtained is perceived as tough by the consumers [4]. China is the top producer of donkey (192,000 tons/year) and mule meat (50,000 tons/year), followed by Niger for donkey meat (8800 tons/year) and Spain with 676 tons/year for mule meat [5].

Several studies demonstrated that donkey meat shows an interesting content of high biological value proteins and low levels of fat, characterized by a healthy fatty acid profile [6,7,8]. Chemical composition and physical traits have been investigated in different horse muscles [9,10] and in donkey muscles [11], while little research has been conducted on the chemical composition and physical traits of mule meat. In fact, mule meat is normally used for the manufacture of salami [12] or other derived product [13], because it is considered tough by the consumers.

The objective of this study was to compare the carcass growth parameters and the physical and chemical characteristics determined in the muscle Longissimus thoracis taken from extensively reared mature male donkeys and mules.

## 2. Materials and Methods

### 2.1. Animals and Diets

The experimental protocol was approved by the Ethical Committee on Animal Experimentation of the University of Camerino. In addition, the authors declare that the trial has been carried out according to the European guidelines regarding ethical use of animals, applying a high standard of veterinary care.

Ten crossbred male donkeys (Martina Franca x Ragusana) and 10 male mules reared in the same farm were slaughtered at an age of 16 ± 1 years in groups of four animals per time within a period of six months, with an average final body weight of 285 ± 11 kg for donkeys and 363 ± 15 kg for mules. The animals were reared for the last three years of their life under grazing conditions on grass pasture during the day, and received in the evening a diet (2 kg/head) based on meadow hay, oats, and carrots, with free access to water.

### 2.2. Slaughter Procedure, Carcass Evaluation, and Sampling

All the animals were weighed before their arrival at a European Community-approved abattoir, according to European Community laws on Animal Welfare in transport (1/2005EC) and the European Community regulation on Animal Welfare for the slaughter of commercial animals (1099/2009EC), where they were electrically stunned and slaughtered by cutting the jugular vein.

All the non-carcass components (skin, head, feet, heart, lungs, liver, spleen, and the entire digestive tract) were removed, then the carcasses were transferred to a cold room at a temperature of 4 °C and stored suspended by the hind legs. After 24 h, the cold carcass weights were recorded, and the dressing percentages were calculated as the cold carcass weight divided by the slaughter weight. From the right side of each carcass, 24 h after slaughtering the muscle Longissimus thoracis (LT) was sampled between the 9th and the 13th rib from each animal; the average sample weight was 250 g/animal. Samples were put in vacuum bags and stored at 4 °C until chemical and physical analysis.

### 2.3. Chemical Analysis

Forty-eight hours after slaughtering, 50 g of meat was taken from each LT sample for the determination of the moisture, protein, fat, and ash contents [14]. For cholesterol determination, lipids were saponified [15]. Glycogen was determined spectrophotometrically (340 nm) using a commercial kit from Sigma (Glucose HK 16–50), according to the procedure published by [16]. The total collagen content was calculated multiplying the hydroxyproline concentration for a conversion factor of 7.14 [17]; the results were expressed in μg/mg wet tissue. The fatty acid composition was determined for both the mule and donkey LT muscles; the total lipids were extracted [18], then the fatty acids methyl esters were obtained [19]. The fatty acid peaks were identified by comparison with the retention times of standard fatty acids. The fatty acids contents were expressed as a percentage of the total fatty acids.

Amino acids determination was performed by HPLC using the AccQ-Fluor reagent kit from Waters (Milford, MA, USA). Amino acids were identified by retention time using an amino acid, standard and Norleucine was added as an internal standard [20]. Macro (Ca, K, Mg, Na, P) and microelements (Cu, Mn, Fe, Zn) were mineralized using the Mileston Ethos 900 microwave, adding 64% nitric acid, hydrogen peroxide, and ultrapure water [21].

### 2.4. Physical Analysis

Color parameters were measured 48 h after slaughter on a fresh surface of the muscles after 1 h of oxygen exposure at room temperature using a Minolta CM-3600 D spectrophotometer (Konica Minolta Holdings Inc., Marunouchi, Chiyoda, Tokyo, Japan), with a measured area diameter of 8 mm, a standard illuminant D65, and an observer angle of 10°. Zero and white calibrations were made with the standard tiles in order to determine the L* (lightness), a* (redness), and b* (yellowness) [22]. After placing the measuring lens on the meat surface, it was turned through 0°, 45°, and 90° (clockwise) to obtain three different reflectance measurements that were later averaged.

Samples for shear force determination, weighting approximately 30 g, were put in a vacuum bag and stored in the cold room at 4 °C for 6 days post slaughter before evaluating the shear force values, performed on three blocks (1.5 cm in length and 1 by 1 cm cross section) for each sample, roasted on a metal tray at an oven temperature of 80 °C to an internal temperature of 73 °C, and monitored with thermocouples. Chops were cooled to room temperature (23 °C) for 30 min. From each sample, 8 cores (1.2 cm in diameter) were sheared with a Warner-Bratzler operating head mounted on an Instron apparatus 4411 (Instron, High Wycombe, UK) and a crosshead speed set at 200 mm/min. The force was applied perpendicularly to the direction of the fibres. The maximum force necessary to break fibres was determined using a Warner-Bratzler device equipped with a triangular-shaped blade mounted an Instron apparatus 4411 (Instron, High Wycombe, UK), with a crosshead speed set at 200 mm/min [22]. The shear force values were expressed in N/cm^2^.

### 2.5. Statistical Analysis

An analysis of variance was used to determine the significance of differences in the values obtained in this study, using the general linear model procedures of the statistical package of SAS [23]. Significant differences were shown when *p* < 0.05.

## 3. Results and Discussion

### 3.1. Carcass Characteristics

The final body weights determined before slaughtering were significantly (*p* < 0.05) higher for mules (Table 1). The donkey cold carcass weight (154 ± 2.35 kg) and dressing percentage (53.7%) were significantly (*p* < 0.05) lower compared to the values determined for mules, which were. Respectively. 212 ± 4.21 kg and 58.4%. Aganga et al. [24] found similar values for donkey dressing percentages (54.5%) in animals slaughtered at 7 years of age; to our knowledge, scientific data regarding mule dressing percentage are not available. French horse breeds show the highest dressing percentages, close to 70%, but this value decreased to below 59% for local autochthonous horse breeds [25,26]; animals belonging to these horse local breeds show final body weights similar to the mules and donkeys used in the present study.

### 3.2. Meat Chemical and Physical Characteristics

The protein content was very similar both in donkey and mule muscle (Table 2). The fat content was significantly (*p* < 0.05) higher in the donkey LT (3.30 g/100 g), while the ash and water content were not statistically different. The protein content determined in the muscle LT taken from both donkey and mule carcasses was very similar to that obtained [27] in muscle LT sampled from Burguete horses slaughtered at 16 months of age (19.9 g/100 g), while the protein content detected [28] in Martina Franca donkeys slaughtered at 18 months was higher (22.3 g/100 g).

The cholesterol content was not significantly affected by the muscle type; the highest amount (68.4 mg/100 g) was observed in donkey muscle LT (Table 2). The cholesterol content was very similar to the value obtained [29] in meat produced by horses slaughtered at an age ranging between 72 and 120 months, and also to the cholesterol content determined in muscle LT taken from Martina Franca donkeys slaughtered at an age of 8 months [30].

The glycogen content (Table 2) was significantly higher (*p* < 0.05) in mule muscle LT (2.03 g/100 g). The values determined in both mule and donkey muscles were markedly higher compared to the glycogen content detected in meat samples taken from Martina Franca donkeys slaughtered at 12 months [30]. The glycogen content in horse and in donkey meat has been detected in previous studies, showing significant differences according to the different muscles examined and to the different slaughtering ages [9].

The amount of total collagen was significantly (*p* < 0.05) higher in the LT (69.3 μg/mg) mule muscle compared to the donkey LT muscle. The values obtained in this study were higher compared to the results determined [11] in the muscle Semitendinosus and in the muscle Semimembranosus in crossbred donkeys slaughtered in Italy at 10 months of age and with a mean final body weight of 126 kg. This difference can be attributed to the different ages of the animals used, as well as different muscle anatomy and muscle usage [31]. In fact, it has been reported [8] that the collagen content in horse muscles vastly increases from 12 to 18 months of age, then remains stable. Consequently, the mules and the donkeys used in this study, slaughtered at a final age of 10 years, showed a higher content of total collagen (Table 2) compared to the values obtained when muscles were sampled in younger animals [32].

Meat color is affected by animal age, sex, and the anatomical location of the muscle [8]. In the present study (Table 2), muscle LT taken from mule carcasses showed a lightness parameter (L* 31.27) significantly (*p* < 0.05) lower than that of the same donkey muscle (L* 37.91). The redness index (a*) was significantly (*p* < 0.05) higher in the mule muscle LT (a* 18.3) compared to the donkey muscle LT (Table 2), while the yellowness index (b*) was not significantly different among the muscles examined.

Lightness (L*) and redness (a*) values are mainly affected by the muscle myoglobin content, which is higher in older animals [33], influencing strongly the meat color, a particularly important factor on which consumer purchasing decisions are based. A red color is associated with freshness also in donkey meat sensory evaluation [4]. The mule muscle LT showed a high redness index (a*) that could be associated with meat color passage from red to brown due to the myoglobin redox state and its muscle content [34]. The results obtained in the present study demonstrate that mule meat color is darker compared to donkey meat.

The results of the tenderness evaluation performed six days after slaughtering in mule and donkey muscles are shown in Table 2. Mule muscle LT reported significantly (*p* < 0.05) higher shear force values compared to the same donkey muscle. The results determined in donkey muscle were remarkably close to the findings obtained for horse meat [35] sampled from 131 mature horses slaughtered at an age close to 10 years and with a mean final body weight of 464 kg. The values of shear force obtained in this study in LT donkey muscle are not perceived as tough by most of the consumers [36], while the shear force value determined in the mule muscle LT is considered the threshold before perceiving the meat as “tough” [37]. A possible strategy to reduce the toughness in mule meat could be marinating the meat during cold storage, and using different organic acids, such lactic acid, malic acid, etc., as demonstrated in horse meat produced by old animals [38].

### 3.3. Fatty Acid Composition

The results obtained for the fatty acid profile for donkey and mule LT muscle are shown in Table 3. No significant differences have been reported comparing mule and donkey muscle LT. In both the muscles examined, oleic acid (C18:1 n-9) was the most represented fatty acid, followed by palmitic acid (C16:0) and then linoleic acid (C18:2). Rumenic acid (C18:2 cis 9, trans 11), the most represented of the conjugated linoleic acid isomers (CLAs), has been detected in different amounts in both the muscles. CLAs are normally found in ruminant meat because they are produced by the biohydrogenation of unsaturated fatty acids performed by rumen bacteria [39]. Among CLAs, rumenic acid is considered very important for human health [40]; this isomer has been previously determined in horse meat obtained from foals slaughtered at 24 months of age [41], while CLAs have never been detected before in the donkey meat lipid profile [42]. Meat produced by mules and donkeys slaughtered at 10 years of age seems to possess a small amount of CLAs, similar to horse meat. Both eicosapentaenoic acid (EPA; C20:5 n-3) and docosahexaenoic acid (DHA; C22:6 n-3) were detected in the two muscles examined; the intake of these essential fatty acids can reduce the risk of coronary heart disease [43]. The results obtained in this study confirmed that donkey meat is a good source of polyunsaturated n-3 fatty acids, with consequent positive effects on consumer health [44].

### 3.4. Mineral and Amino Acid Content

The mineral content is shown in Table 4. Within macro elements, no significant differences were detected among the mule and donkey muscles. Potassium is the most abundant macro element in both the examined muscles. This mineral can be considered essential for life; since it is involved in the insulin secretion pathways, it is necessary for muscle contraction and, together with sodium, it helps to regulate osmotic pressure in the cell [45]. The second macro mineral determined in mule and donkey meat is phosphorous, with values higher compared to those reported in beef, pork, or lamb [45]. Considering the micro elements (Table 4), the iron content was significantly (*p* < 0.05) higher in the muscle LT (2.92 mg/100 g) sampled on the mule carcass, while zinc was significantly (*p* < 0.05) higher in the muscle LT taken from donkey carcass (5.25 mg/100 g). The iron in meat is mostly haem iron, which is well-absorbed, similar to zinc absorption, which is greater in a diet high in animal protein [46]. The values of iron and zinc contents obtained in this experiment were remarkably close to those determined in donkey meat produced by the Martina Franca breed [7].

The amino acid content in donkey and mule LT muscle is shown in Table 5. Both the donkey and mule muscles provide all the essential amino acids; in this group, leucine and lysine were the most represented in both the mule and donkey LT muscle, followed by arginine, confirming the results obtained in previous studies performed both on horse [26] and donkey meat [4]. Donkey muscle LT showed significantly (*p* < 0.05) higher contents of methionine, valine, and essential amino acids (52.2% of the total amino acids) compared to the mule LT muscle. In the non-essential amino acid fraction, glutamine showed the highest content, followed by aspartic acid, alanine, and glycine, confirming the results obtained by Polidori et al. [30]. Donkey and mule meats appear to be a good source of proteins with a high biological value, due to their essential amino acid content and the absence of limiting amino acids [46].

## 4. Conclusions

Consumer demand for alternative red meats has increased. Both donkey and mule LT muscle contain a relatively low fat and cholesterol content, an interesting amount of essential fatty acids, and CLA. Both donkey and mule LT muscle is rich in protein and possesses many essential amino acids and important minerals. Donkey breeding could represent a meat production system characterized by low environmental impact, while meat produced by mules, perceived as tough, can be used for preparing salami and other derived products. Further studies are required in order to better understand the effects of different feeding strategies on donkey meat quality, and the influence of an early slaughter age on mule meat tenderness.

## Figures and Tables

**Table 1:** Mule and donkey carcass characteristics (means ± SE).

Performance Data	Mule (*n* = 10)	Donkey (*n* = 10)
Final Body Weight (kg)	363 ± 15 ^a^	285 ± 11 ^b^
Cold Carcass Weight (kg)	212 ± 4.21 ^a^	154 ± 2.35 ^b^
Cold Dressing (%)	58.4 ± 0.82 ^a^	53.7 ± 0.93 ^b^

Different letters on the same row show differences that are statistically significant (^a,b^: *p* < 0.05; SE: Standard Error).

**Table 2:** Chemical and physical characteristics of muscle Longissimus thoracis (means ± SE) taken from mule and donkey carcasses.

	Mule (*n* = 10)	Donkey (*n* = 10)
**Chemical composition**		
Moisture (%)	74.3 ± 3.18	74.2 ± 2.94
Protein (%)	20.1 ± 1.04	19.9 ± 1.05
Fat (%)	2.64 ± 0.77 ^a^	3.30 ± 0.56 ^b^
Ash (%)	0.95 ± 0.06	1.03 ± 0.04
Glycogen (%)	2.03 ± 0.12 ^a^	1.61 ± 0.10 ^b^
Cholesterol (mg/100 g)	59.2 ± 1.99	68.4 ± 2.18
Total collagen (μg/mg)	69.3 ± 3.32 ^a^	47.8 ± 3.49 ^b^
**Color parameters**		
Lightness (L*)	31.27 ± 2.43 ^a^	37.91 ± 2.63 ^b^
Redness (a*)	18.3 ± 0.40 ^a^	11.7 ± 0.39 ^b^
Yellowness (b*)	9.94 ± 0.24	8.30 ± 0.27
**Shear force value**		
WBSF 6 days (N/cm^2^)	58.3 ± 4.23 ^a^	49.4 ± 3.43 ^b^

Different letters on the same row show differences that are statistically significant (^a,b^: *p* < 0.05; SE: Standard Error). WBSF: Warner-Bratzler Shear Force.

**Table 3:** Fatty acid composition (% total fatty acids) determined in the Longissimus thoracis muscles of mules and donkeys (means ± SE).

Fatty Acid	Mule (*n* = 10)	Donkey (*n* = 10)
C10:0 Capric acid	0.09 ± 0.01	0.06 ± 0.01
C12:0 Lauric acid	0.24 ± 0.10	0.16 ± 0.09
C14:0 Myristic acid	2.61 ± 0.55	2.66 ± 0.52
C14:1 Myristoleic acid	0.20 ± 0.09	0.19 ± 0.07
C15:0 Pentadecanoic acid	0.18 ± 0.08	0.16 ± 0.04
C15:1 Pentadecenoic acid	0.06 ± 0.001	0.09 ± 0.001
C16:0 Palmitic acid	25.9 ± 1.49	25.2 ± 1.41
C16:1 n-9 Palmitoleic acid	4.34 ± 0.83	4.25 ± 0.74
C17:0 Margaric acid	0.50 ± 0.05	0.56 ± 0.04
C17:1 Heptadecenoic acid	0.36 ± 0.09	0.46 ± 0.10
C18:0 Stearic acid	5.39 ± 0.87	5.69 ± 0.81
C18:1 n-9 Oleic acid	30.4 ± 2.81	30.2 ± 2.88
C18:2 n-6 Linoleic acid	23.2 ± 2.70	23.7 ± 2.77
C18:2 cis9,trans11 CLA Rumenic acid	0.06 ± 0.001	0.05 ± 0.001
C18:3 n-6 gamma-Linolenic acid	0.04 ± 0.005	0.04 ± 0.003
C18:3 n-3 alpha-Linolenic acid	3.28 ± 0.61	2.95 ± 0.47
C20:0 Arachidic acid	0.06 ± 0.002	0.07 ± 0.006
C20:1 Paullinic acid	0.04 ± 0.008	0.03 ± 0.005
C20:4 n-6 Arachidonic acid	2.19 ± 0.72	2.48 ± 0.65
C20:5 n-3 EPA Eicosapentaenoic acid	0.12 ± 0.001	0.09 ± 0.003
C21:0 Henecoisilic acid	0.04 ± 0.004	0.02 ± 0.003
C22:0 Docosanoic acid	0.17 ± 0.07	0.21 ± 0.06
C22:2 n-6 Cetoleic acid	0.14 ± 0.02	0.19 ± 0.01
C22:4 n-6 Docosatetraenoic acid	0.07 ± 0.006	0.08 ± 0.005
C22:5 n-3 DPA Docosapentaenoic acid	0.26 ± 0.05	0.30 ± 0.06
C22:6 n-3 DHA Docosaexaenoic acid	0.07 ± 0.004	0.07 ± 0.005
C24:0 Tetracosanoic acid	0.05 ± 0.009	0.04 ± 0.004
C24:1 Nervonic acid	0.03 ± 0.006	0.02 ± 0.008
∑ SFA	35.23 ± 3.25	34.83 ± 3.38
∑ MUFA	35.43 ± 3.34	35.24 ± 3.42
∑ PUFA	29.36 ± 2.63	29.95 ± 2.59
PUFA/SFA	0.83	0.86
∑ n-6	25.6	26.5
∑ n-3	3.73	3.41
n-6/n-3	6.86	7.77

SE: Stabdard Errur; SFA: Saturated Fatty Acids; MUFA: Mono Unsaturated Fatty Acids; PUFA: Poly Unsaturated Fatty Acids; ∑ n-6; Sum omega 6 fatty acids; ∑ n-3: Sum omega 3 fatty acids.

**Table 4:** Mineral content (mg/100 g wet tissue) determined in mule and donkey Longissimus thoracis muscle (means ± SE).

Mineral	Mule (*n* = 10)	Donkey (*n* = 10)
**Macroelements**		
Ca	3.30 ± 0.58	3.45 ± 0.44
K	310 ± 24.3	320 ± 28.6
Mg	23.3 ± 1.28	21.5 ± 1.18
Na	70.6 ± 3.38	73.7 ± 3.71
P	211.8 ± 11.1	207.6 ± 10.7
**Microelements**		
Cu	0.23 ± 0.05	0.24 ± 0.06
Mn	0.03 ± 0.004	0.02 ± 0.007
Fe	2.92 ± 0.41 ^a^	2.47 ± 0.39 ^b^
Zn	4.12 ± 0.13 ^a^	5.25 ± 0.18 ^b^

Different letters on the same row show differences statistically significant (^a,b^: *p* < 0.05; SE: Standard Error).

**Table 5:** Amino acid composition (g/100 g muscle) of mule and donkey Longissimus thoracis muscle (means ± SE).

Amino Acid	Mule (*n* = 10)	Donkey (*n* = 10)
**Essential**		
Arginine	1.17 ± 0.26	1.25 ± 0.13
Histidine	0.89 ± 0.20	0.93 ± 0.17
Isoleucine	0.90 ± 0.22	1.04 ± 0.33
Leucine	1.52 ± 0.57	1.61 ± 0.27
Lysine	1.56 ± 0.26	1.62 ± 0.25
Methionine	0.48 ± 0.23 ^a^	0.69 ± 0.28 ^b^
Phenylalanine	0.84 ± 0.27	0.87 ± 0.23
Threonine	0.85 ± 0.22	0.99 ± 0.25
Tryptophan	0.29 ± 0.16	0.32 ± 0.11
Valine	0.95 ± 0.46 ^a^	1.09 ± 0.51 ^b^
**Non Essential**		
Alanine	1.18 ± 0.15	1.29 ± 0.31
Aspartic acid	1.81 ± 0.34	1.84 ± 0.47
Cystine	0.15 ± 0.03	0.19 ± 0.07
Glutamine	2.89 ± 0.85	3.02 ± 0.88
Glycine	1.04 ± 0.48	0.91 ± 0.55
Proline	0.90 ± 0.34	0.93 ± 0.46
Serine	0.70 ± 0.31	0.78 ± 0.32
Tyrosine	0.66 ± 0.26	0.64 ± 0.37
Total AA	18.7 ± 3.12	19.9 ± 3.23
Essential AA (%)	9.45 ± 2.68 (50.1%) ^a^	10.4 ± 2.46 (52.2%) ^b^

Different letters on the same row show differences that are statistically significant (^a,b^: *p* < 0.05; SE: Standard Error); AA: Amino Acids.
